# Supplementary material for: Erbium-Doped GQD-Embedded Coffee-Ground-Derived Porous Biochar for Highly Efficient Asymmetric Supercapacitor
Source: Nanomaterials (Basel). 2022 Jun 6;12(11):1939. doi: 10.3390/nano12111939 (PMC9182556; doi:10.3390/nano12111939)
Supplement: Supplementary file 1 [file nanomaterials-12-01939-s001.zip › nanomaterials-1748330-supplementary.pdf]

nanomaterials

MDPI

Supplementary Materials

Erbium-Doped GQD-Embedded Coffee-Ground-Derived Porous Biochar for Highly Efficient Asymmetric Supercapacitor

Thi Ai Ngoc Bui [1], Trung Viet Huynh [2], Hai Linh Tran [1] and Ruey-an Doong [2,*]

[1] Department of Biomedical Engineering and Environmental Sciences, National Tsing Hua University, 101, Sec. 2, Kuang Fu Road, Hsinchu 30013, Taiwan; buiaingoc.ep03g@g2.nctu.edu.tw (T.A.N.B.); tranlinhhai@gmail.com (H.L.T.)

[2] Institute of Analytical and Environmental Sciences, National Tsing Hua University, 101, Sec. 2, Kuang Fu Road, Hsinchu 30013, Taiwan; htviet1993@gmail.com

* Correspondence: radoong@mx.nthu.edu.tw

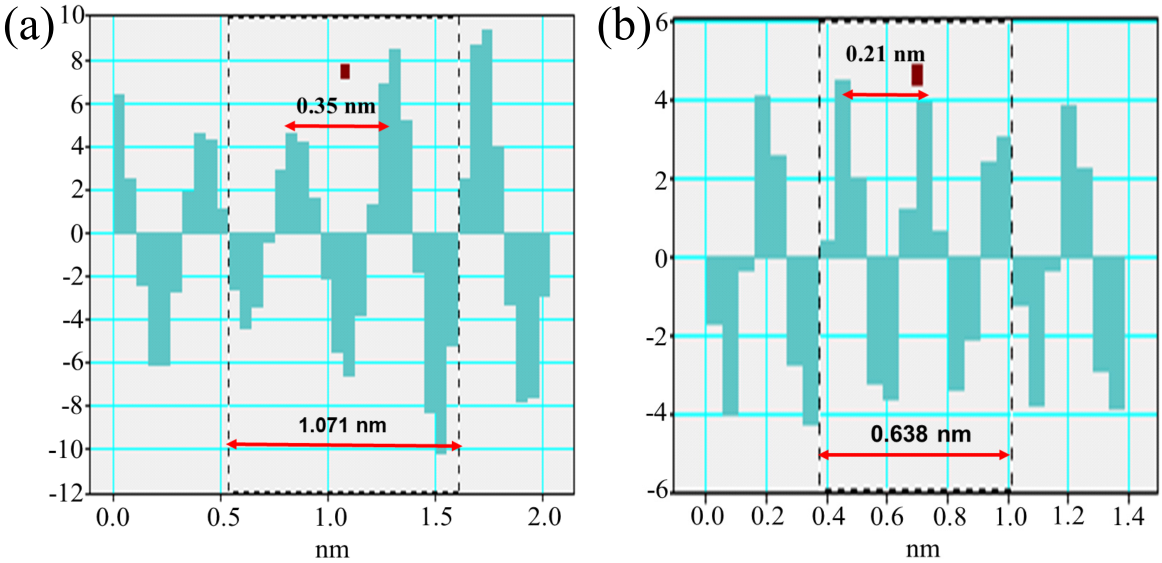

Figure S1. Lattice spacings of (**a**) pure HPB and (**b**) Er 10-GQD/HPB.

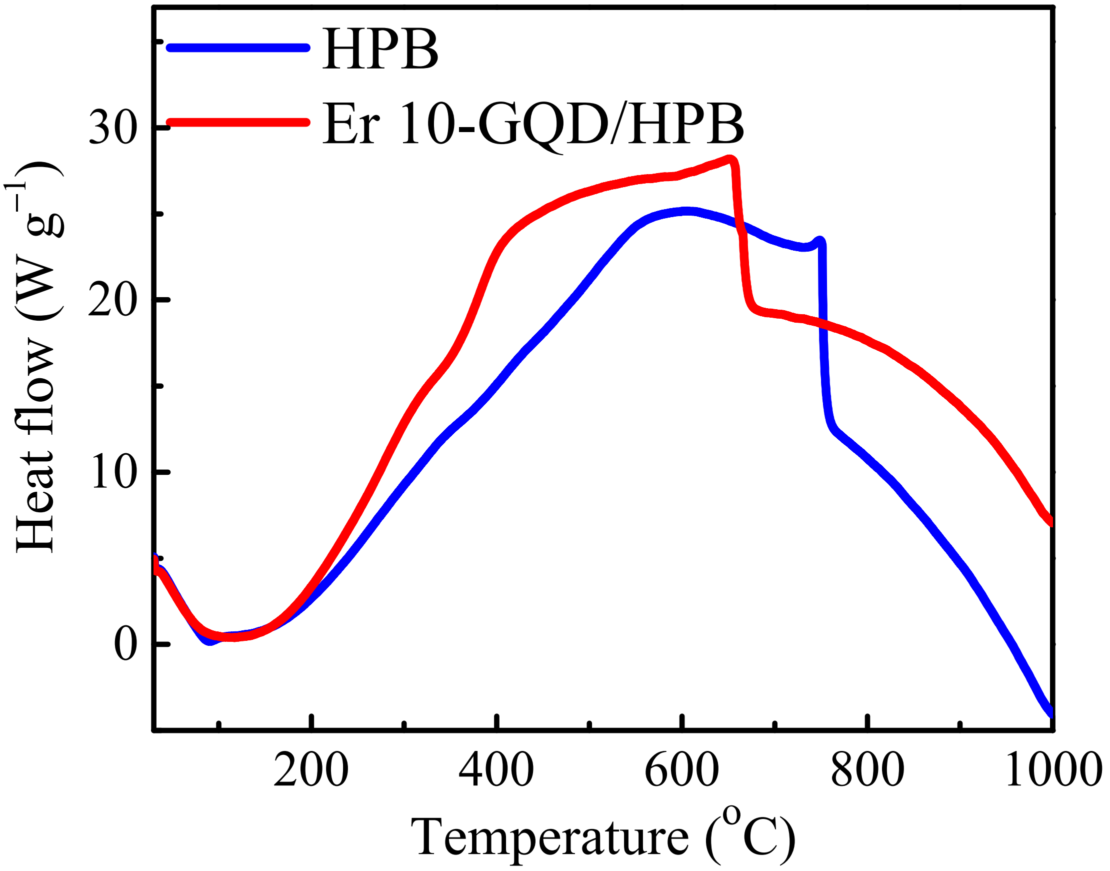

Figure S2. The differential scanning calorimetry (DSC) of HPB and Er 10-GQD/HPB.

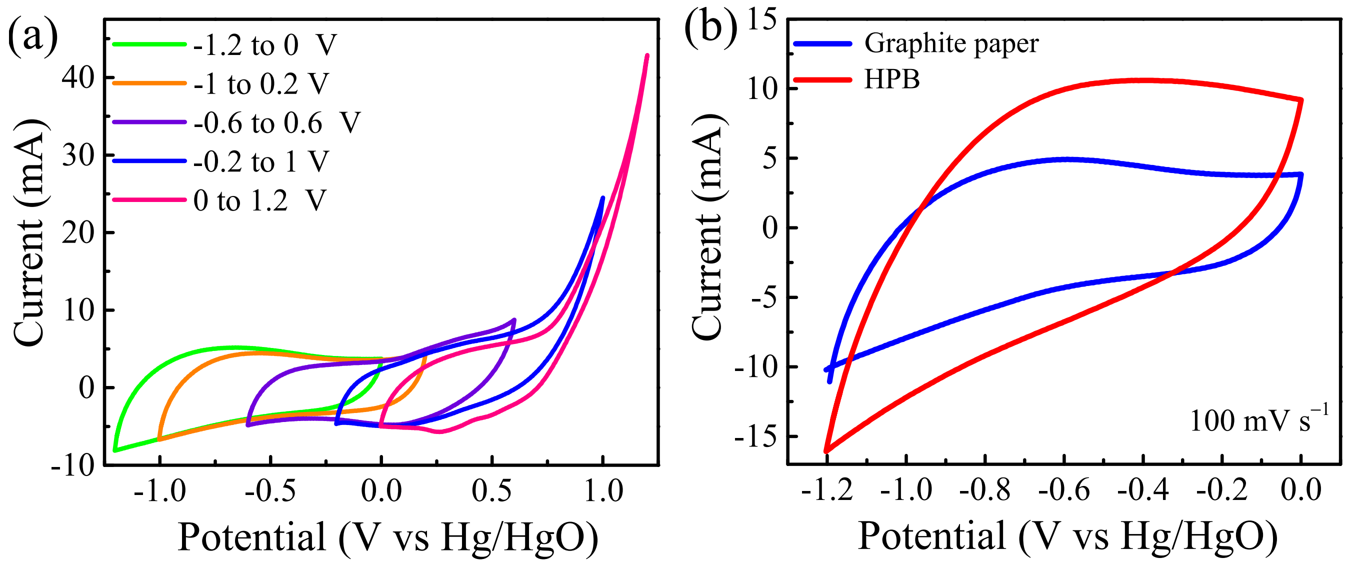

Figure S3. CV curves of (**a**) graphite paper and (**b**) HPB and graphite paper at 100 mV s^{-1} in different voltage windows of −1.2–1.0 V.

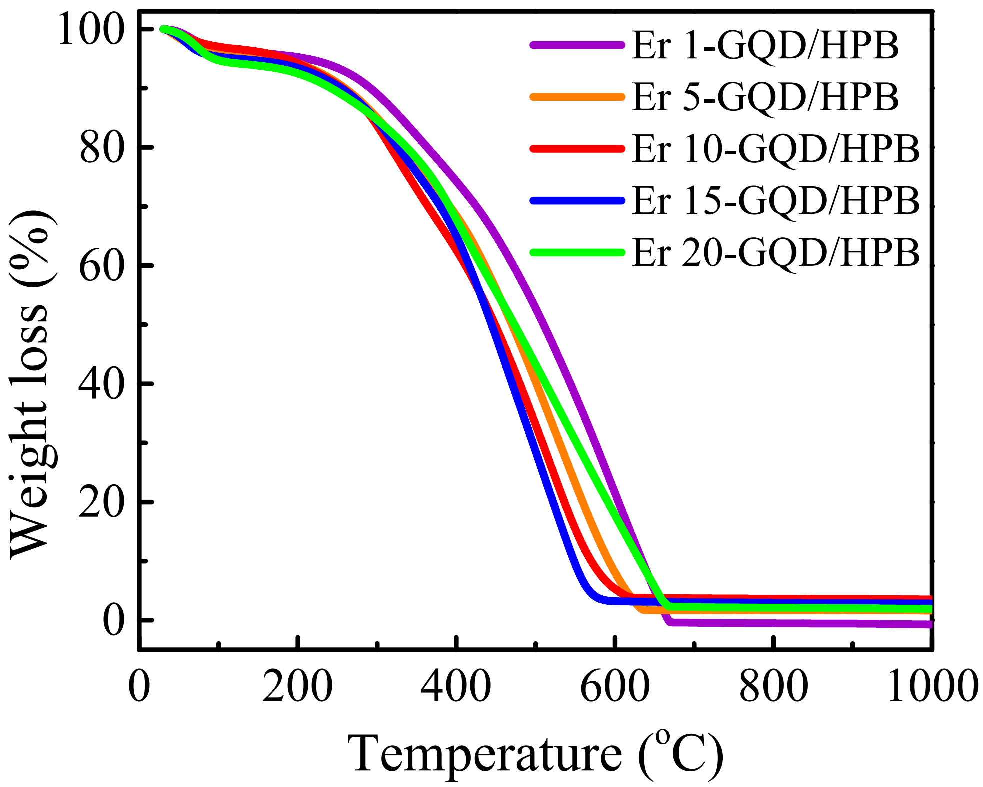

Figure S4. TGA curves of Er-GQD/HPB nanocomposite in various Er loading from 1 to 20 mM.

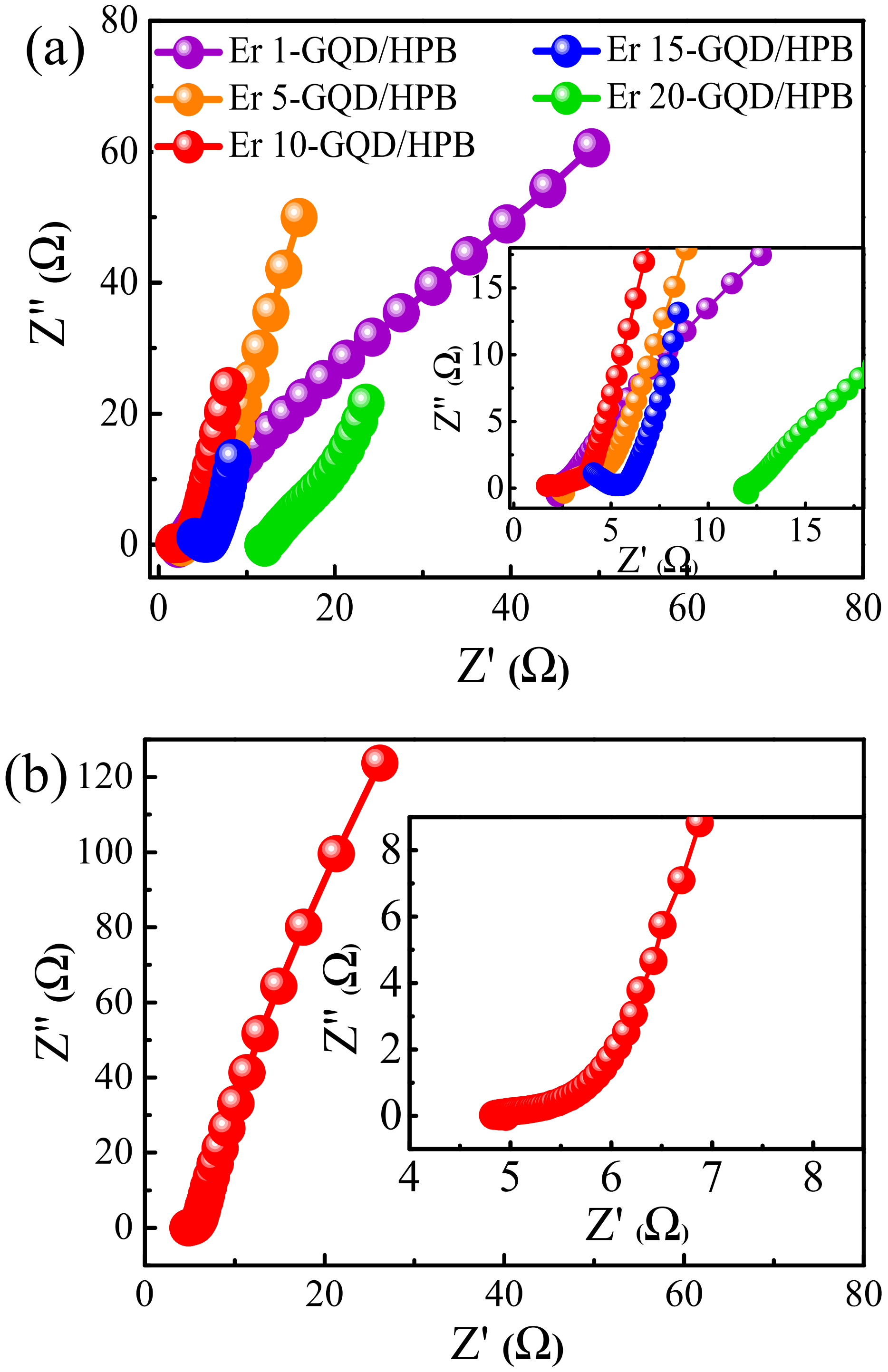

Figure S5. Nyquist plots of (**a**) Er-GQD/HPB at different Er loadings of 1–20 mM and (**b**) pure HPB.

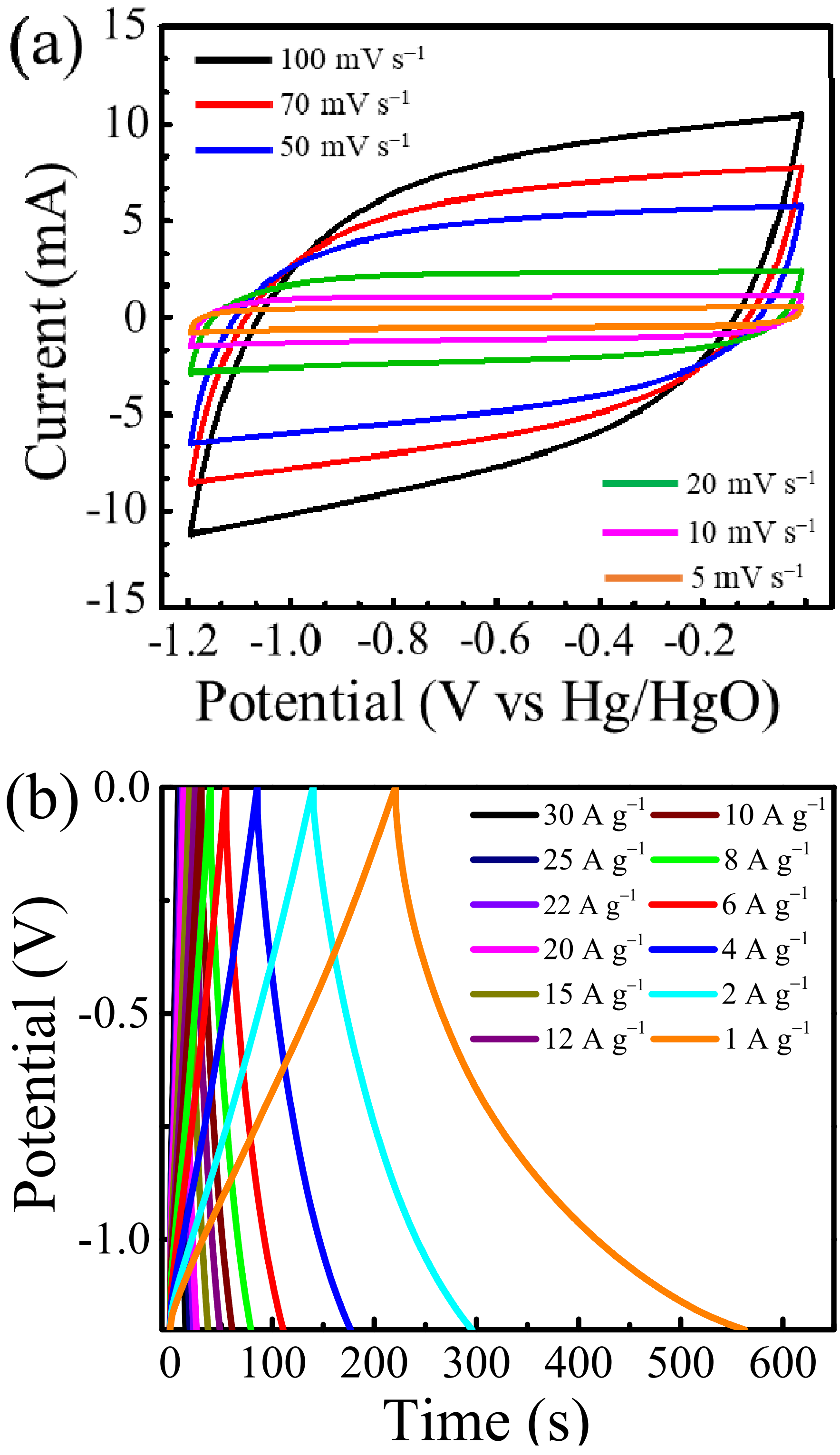

Figure S6. (**a**) CV curves and (**b**) GCD of pure HPB.

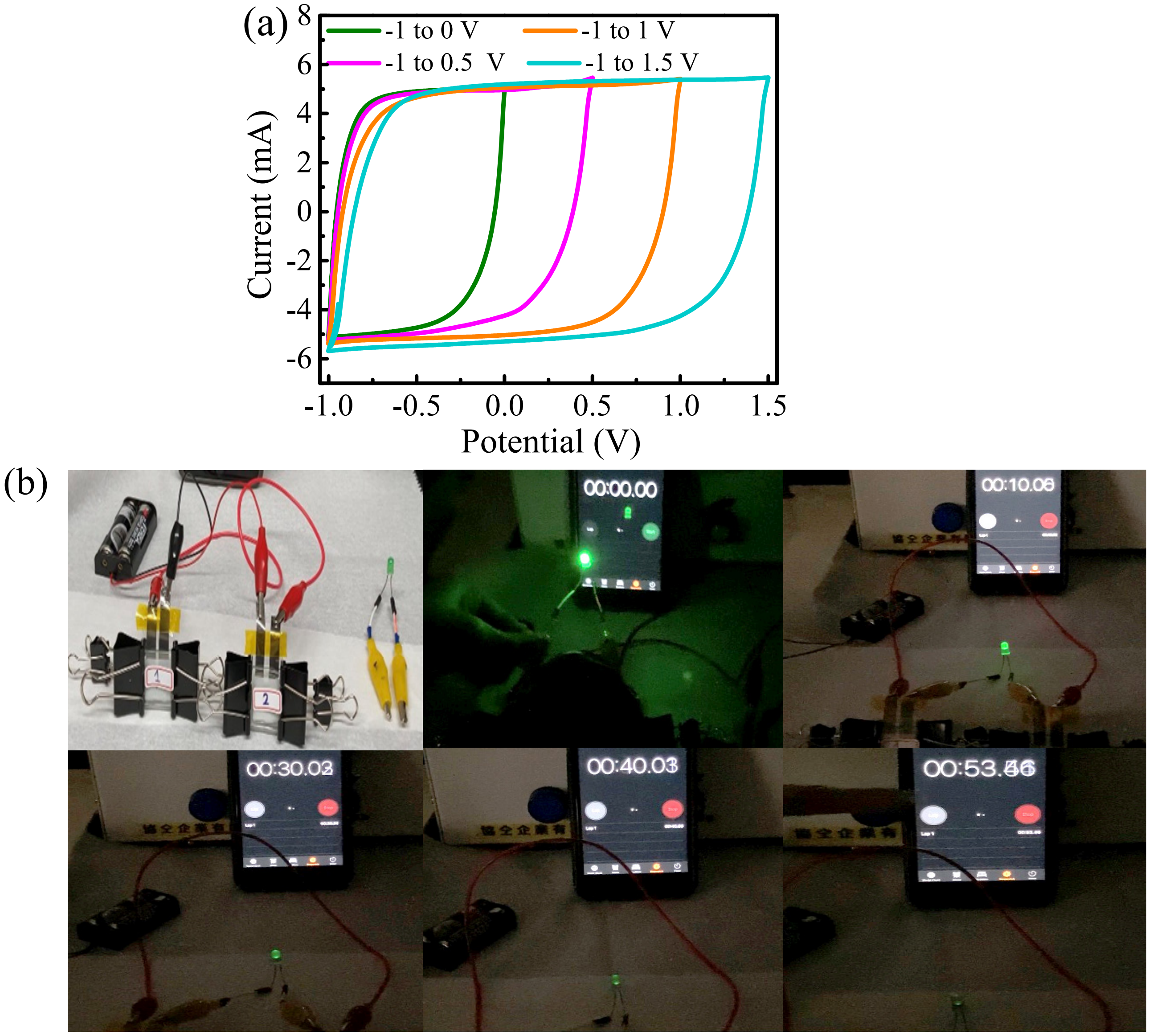

Figure S7. (**a**) CV curves of Er 10-GQD/HPB||HPB in different voltage windows at 100 mV s^{-1} and (**b**) demonstration of lighting of LED powered by Er 10-GQD/HPB||HPB.
